# Supplementary material for: Robot-Assisted Radical Prostatectomy by Lateral Approach: Technique, Reproducibility and Outcomes
Source: Cancers (Basel). 2023 Nov 16;15(22):5442. doi: 10.3390/cancers15225442 (PMC10670058; doi:10.3390/cancers15225442)
Supplement: Supplementary file 1 [file cancers-15-05442-s001.zip › cancers-2627367-supplementary.pdf]

Supplementary material

Table S1. pT3 stage in radical prostatectomy specimens *n = 513 patients submitted to robot assisted radical prostatectomy by lateral approach*

	Surgeon 1 (n= 289)	*Surgeon 2 (n= 224)*
pT3a	*38 (13.14%)*	*47 (20,98%)*
PSM	*21 (55.26%)*	*18 (38.29%)*
sPSM	*4 (10.52%)*	*3 (6.38%)*
Persistence	*1 (2.63%)*	*2 (4.24%)*
BR	*8 (21.05%)*	*7 (14.8%)*
pT3b	*25 (8,65%)*	*14 (6.25%)*
PSM	*8 (32%)*	*7 (50%)*
sPSM	*2 (8%)*	*3 (21.42%)*
Persistence	*3 (12%)*	*2 (14,28)*
BR	*5 (20%)*	*4 (28,57%)*
